# Revealing novel pyroptosis-related therapeutic targets for sepsis based on machine learning

**DOI:** 10.1186/s12920-023-01453-7

**Published:** 2023-02-10

**Authors:** Ying Chen, Xingkai Wang, Jiaxin Wang, Junwei Zong, Xianyao Wan

**Affiliations:** 1 Department of Critical Care Medicine, The First Affiliated Hospital of Dalian Medical University, Dalian, Liaoning China; 2 Department of Respiratory Medicine, The Second Affiliated Hospital of Dalian Medical University, Dalian, Liaoning China; 3 Department of Orthopedics, The First Affiliated Hospital of Dalian Medical University, Dalian, Liaoning China; 4 Institute (College) of Integrative Medicine, Dalian Medical University, Dalian, Liaoning China

**Keywords:** Sepsis, WGCNA, Pyroptosis, Machine learning, Prediction model

## Abstract

**Background:**

Sepsis is one of the most lethal diseases worldwide. Pyroptosis is a unique form of cell death, and the mechanism of interaction with sepsis is not yet clear. The aim of this study was to uncover pyroptosis genes associated with sepsis and to provide early therapeutic targets for the treatment of sepsis.

**Methods:**

Based on the GSE134347 dataset, sepsis-related genes were mined by differential expression analysis and **weighted gene coexpression network analysis (WGCNA)**. Subsequently, the sepsis-related genes were analysed for enrichment, and a protein‒protein interaction (PPI) network was constructed. We performed unsupervised consensus clustering of sepsis patients based on 33 pyroptosis-related genes (PRGs) provided by prior reviews. We finally obtained the PRGs mostly associated with sepsis by machine learning prediction models combined with prior reviews. The GSE32707 dataset served as an external validation dataset to validate the model and PRGs via receiver operating characteristic (ROC) curves. The NetworkAnalyst online tool was utilized to create a ceRNA network of lncRNAs and miRNAs around PRGs mostly associated with sepsis.

**Results:**

A total of 170 genes associated with sepsis and 13 hub genes were acquired by WGCNA and PPI network analysis. The results of the enrichment analysis implied that these genes were mainly involved in the regulation of the inflammatory response and the positive regulation of bacterial and fungal defence responses. The prolactin signalling pathway and IL-17 signalling pathway were the primary enrichment pathways. Thirty-three PRGs can effectively classify septic patients into two subtypes, implying that there is a reciprocal relationship between sepsis and pyroptosis. Eventually, NLRC4 was considered the PRG most strongly associated with sepsis. The validation results of the prediction model and NLRC4 based on ROC curves were 0.74 and 0.67, respectively, both of which showed better predictive values. Meanwhile, the ceRNA network consisting of 6 lncRNAs and 2 miRNAs was constructed around NLRC4.

**Conclusion:**

NLRC4, as the PRG mostly associated with sepsis, could be considered a potential target for treatment. The 6 lncRNAs and 2 miRNAs centred on NLRC4 could serve as a further research direction to uncover the deeper pathogenesis of sepsis.

**Supplementary information:**

The online version contains supplementary material available at (10.1186/s12920-023-01453-7).

## Introduction

Sepsis manifests as signs of infection in conjunction with acute organ dysfunction [1]. The high mortality rate due to severe sepsis remains a serious problem despite the increasing understanding of the pathogenesis and the continuous advances in modern treatment techniques, such as appropriate antibiotics, aggressive resuscitation and organ support [2, 3]. Therefore, there is an urgent need to search for an effective treatment for sepsis patients to improve therapeutic efficacy and prognosis.

Pyroptosis is a specific form of cell death leading to loss of plasma membrane integrity, which is induced by the activation of sensors using the inflammasome [4]. Pyroptosis can be triggered by microbial infections, and proper pyroptosis can protect multicellular host organisms from bacterial and microbial infections [5]. However, excessive pyroptosis can lead to massive inflammatory reactions, such as septic shock and multiorgan failure [6]. Although it has been shown that there is a correlation between pyrexia and sepsis, a specific regulatory mechanism is lacking to elucidate the relationship of both.

In this study, we utilized multiple bioinformatics methods to explore genes associated with sepsis. The PRGs mostly related to sepsis were finally clarified by combining machine learning algorithms. We investigated the genetic connection between pyroptosis and sepsis. The PRGs associated with sepsis could be employed as biomarkers for disease diagnosis and therapy monitoring, as well as a reference for early therapeutic targets for sepsis. Long noncoding RNAs (lncRNAs), a type of nonprotein transcript, are involved in messenger RNA (mRNA) splicing and maturation and mRNA stabilization [6, 7]. It has been demonstrated that lncRNAs have a nonnegligible regulatory role in the pathophysiological mechanisms and organismal dysfunction of sepsis [8]. Thus, we constructed a ceRNA network of lncRNAs around the PRGs [9].

## Methods

### Data retrieval and processing

**Fig. 1:**
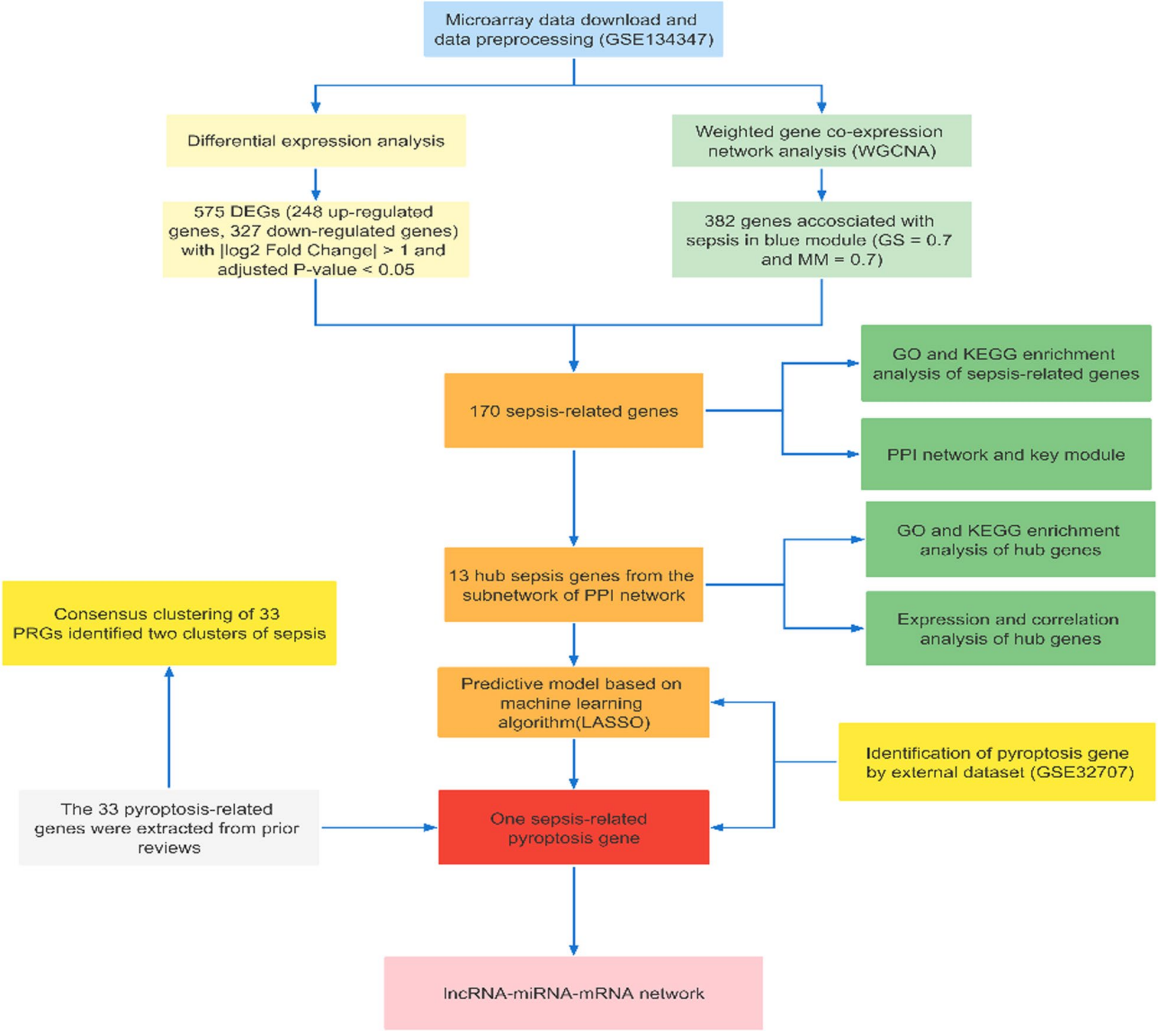
Workflow chart of data preparation, processing, analysis, and validation

We retrieved the dataset using the search terms "(((Expression profiling by array [Filter]) AND Homo sapiens [Organism]) AND blood [Sample Source]) AND sepsis" based on the Gene Expression Omnibus (GEO) (https://www.ncbi.nlm.nih.gov/geo/) database, which is an open-source database consisting of a large amount of tumour or nontumor data. Two suitable datasets (GSE134347 and GSE32707) were obtained and normalized for correction by applying the “sva” R package. Fifty-nine and sixty-nine samples were excluded from the two datasets that were not relevant to this study. Thirty-three genes associated with PRGs were available based on prior reviews (Additional file 4: Table S4). Detailed information on the GEO datasets is listed in Table 1. The flow diagram of the study is shown in Fig. 1.

**Table 1:** Details of the datasets

Dataset	Platform	Count	Sepsis	Healthy	Others
GSE134347	GPL17586 [HTA-2_0] Affymetrix Human Transcriptome Array 2.0 [transcript (gene) version]	298	156	83	59
GSE32707	GPL10558 Illumina HumanHT-12 V4.0 expression beadchip	144	41	34	69

### Identification of DEGs

The identification of differentially expressed genes (DEGs) facilitates the distinguishing of different body state conditions as well as the understanding of gaps between them at the genetic level. The expression matrix, extracted from healthy and sepsis groups, was collated and subjected to differential expression analysis by the “limma” R package. Genes with |log2 Fold Change|> 1 and *P value* < 0.05 were considered DEGs. Meanwhile, these genes were visualized in the form of a volcano map and heatmap by the "ggplot2" and "pheatmap" R packages.

### Weighted gene coexpression network analysis (WGCNA)

The “WGCNA” package was employed for weighted analysis to identify coexpression modules associated with sepsis [10]. First, we chose the optimal soft threshold to construct the adjacency matrix by a calculation and converted it into a topological overlap matrix (TOM). Subsequently, we constructed different modules based on a hierarchical clustering approach and randomly assigned colours to each module, with the difference in colour representing the difference in relevance. The genes in these modules were considered sepsis-related module genes. The genes located in the most relevant block of sepsis were used for subsequent correlation analysis.

### GO and KEGG pathway enrichment analysis of the sepsis-related genes

Genes jointly belonging to the sepsis-related module genes with DEGs were regarded as sepsis-related genes. Genetic enrichment analysis, Gene Ontology (GO) and the Kyoto Encyclopedia of Genes and Genomes (KEGG) were used to measure the distribution trend of genes in a phenotype-related gene table to evaluate their contribution to phenotype. Hence, GO and KEGG enrichment analyses of these genes were executed using the database for annotation, visualization, and integrated discovery (DAVID: https://@@@@@@@david.ncifcrf.gov) (11). The results were visualized using the “ggplot2” package.

### Analysis of the protein‒protein interaction network and hub genes

We applied the Search Tool for the Retrieval of Interacting Genes (STRING) (https://string-db.org/) database to perform protein‒protein interaction (PPI) analysis with the aim of exploring the interconnections between proteins. Subsequently, the raw PPI network was downloaded and built through Cytoscape, a widely used visualization tool. Screening of hub genes from sepsis-associated genes based on overlapping genes was done using multiple algorithms of CytoHubba, a plugin of Cytoscape. Then, the degree of correlation and association between hub genes was visualized by the "corrplot" package. To explore the main mechanisms of hub genes in the pathogenesis of sepsis and their associated pathways of action, we performed functional enrichment analysis using the R package “clusterProfiler”, and FDR < 0.05 was considered significant.

### Unsupervised consensus clustering

We applied an unsupervised clustering approach for subtyping sepsis samples based on 33 PRGs provided by prior reviews, and the algorithm was executed with the ConsensusClusterPlus R package. The number of Clusters k was set from 2 to 9. The cumulative distribution function (CDF) and the area under the CDF curve were used to determine the optimal number of clusters. Subsequently, we identified the clustering results with principal component analysis (PCA).

### Identification and validation of pyroptosis-related genes

The R package “caret” was used to build the least absolute shrinkage and selection operator (LASSO) model, an approved machine learning algorithm, for screening the key genes closely related to sepsis from hub genes. The external dataset GSE32707 was utilized to validate the accuracy of the model in the form of ROC curves, and an AUC value greater than 0.65 was recognized as having a better accuracy. By integrating the key genes obtained above as well as 33 pyroptosis genes, we obtained the pyroptosis genes most strongly associated with sepsis. Finally, the NetworkAnalyst online tool (https://www.networkanalyst.ca/) was used to construct a lncRNA network to gain more insights into the role played by pyroptosis genes in sepsis.

## Results

### Data preprocessing and DEG screening between healthy and sepsis samples

**Fig. 2:**
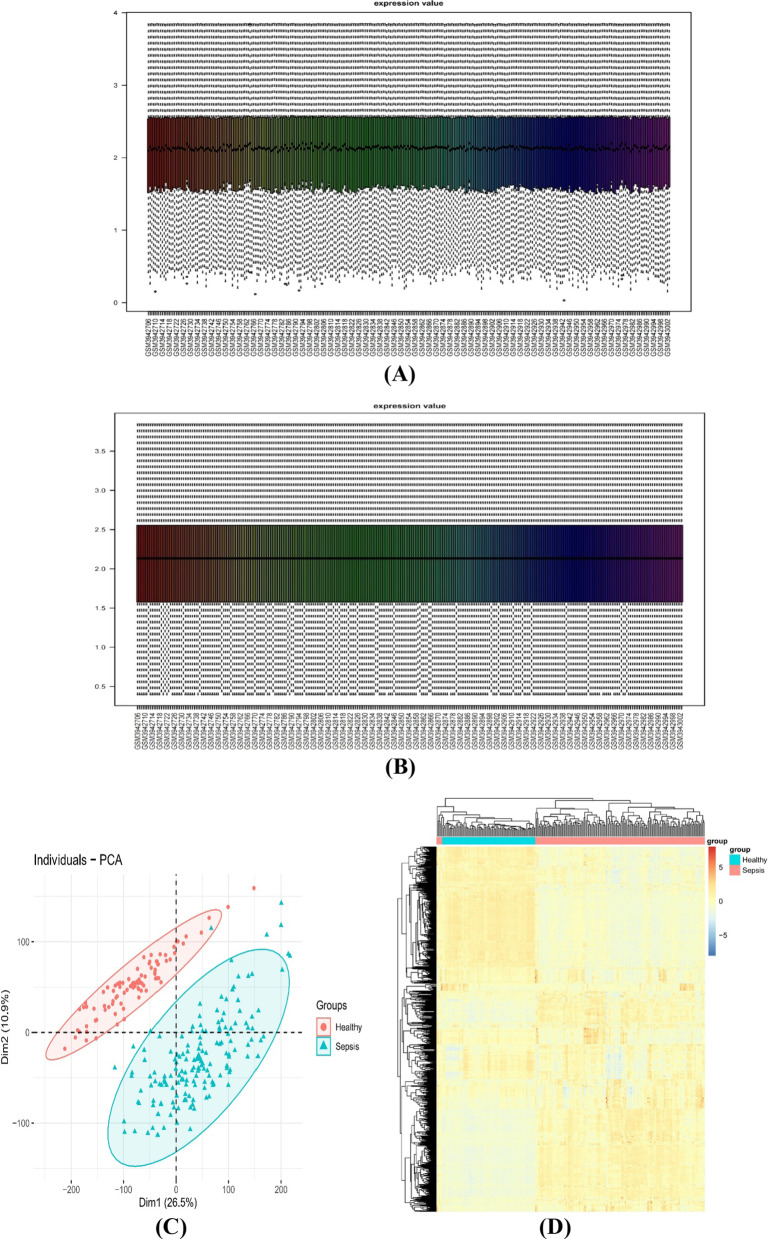

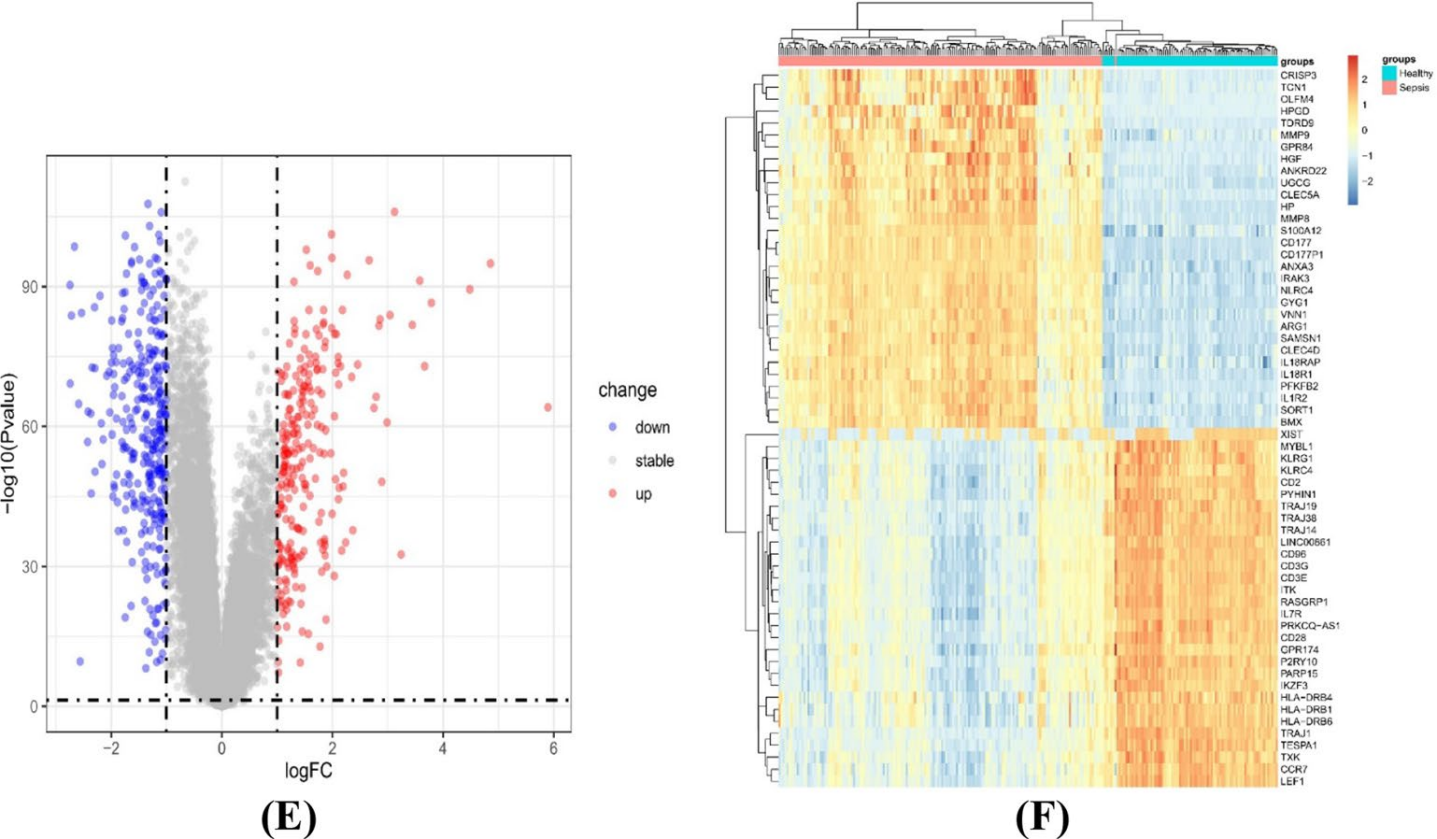
Data preprocessing and DEG screening. **A**, **B** Before & after data normalization. **C** PCA: The farther the two samples are from each other, the greater the difference is between the two samples in gene expression patterns. **D** Heatmap: Gene expression differed between the samples of the two groups. **E** The volcano plot of DEGs: The red points represent upregulated genes, and blue points represent downregulated genes. **F** The heatmap of DEGs: The upregulated genes are shown in red, and downregulated genes are shown in blue

The results of the normalization of the GSE134347 expression data in 83 healthy and 156 sepsis groups are shown in Fig. 2A and 2B. As shown in the PCA results (Fig. 2C), the standardized genes demonstrated by the heatmap (Fig. 2D) could clearly distinguish the healthy samples from the sepsis samples and facilitate further analysis. We screened 575 DEGs based on |log2 Fold Change|> 1 and *P value* < 0.05, and duplicate gene symbols were deleted (Fig. 2E). The heatmap showed 30 upregulated and 30 downregulated genes (Fig. 2F). The details of the DEGs are provided in Additional file 1: Table S1.

### Identification of core modules by WGCNA

**Fig. 3:**
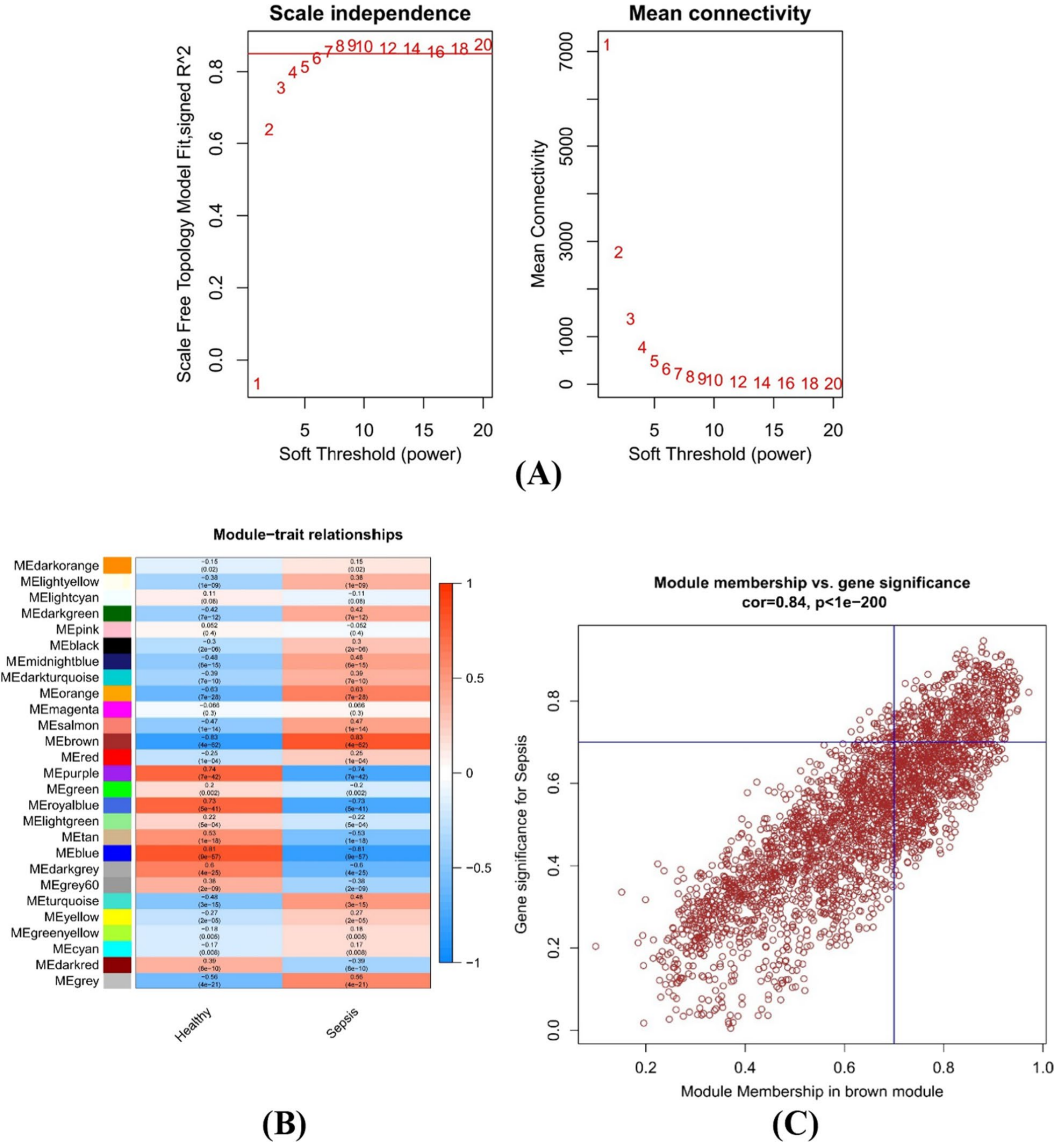
Identification of core modules by weighted gene coexpression network analysis (WGCNA). **A** Left: Analysis of the scale-free index for various soft-threshold powers (β). Right: Analysis of the mean connectivity for various soft-threshold powers. **B** The correlation of genes with sample modules is demonstrated by a heatmap. **C** The relevance of members in the brown module and sepsis

We analysed the critical gene modules closely related to sepsis using the WGCNA algorithm. We screened the soft power β = 6 and the scale-free R^2^ = 0.85 as the most suitable parameters to construct a scale-free network (Fig. 3A). In total, we identified 27 colour modules with different correlations with sepsis. Finally, the brown module exhibited the strongest relationship with sepsis, which included 2,955 genes, r = 0.83, P = 4e − 62 (Fig. 3B). The relationship between modules and disease status was exhibited by the modular significance (MS). Gene significance (GS) was described as the correlation between a gene and clinical phenotype. A total of 382 genes (Additional file 1: Table S1) that they were mostly associated with were screened from this module based on GS = 0.7 and MM = 0.7 (Fig. 3C).

### Genes and pathway enrichment analysis

**Fig. 4:**
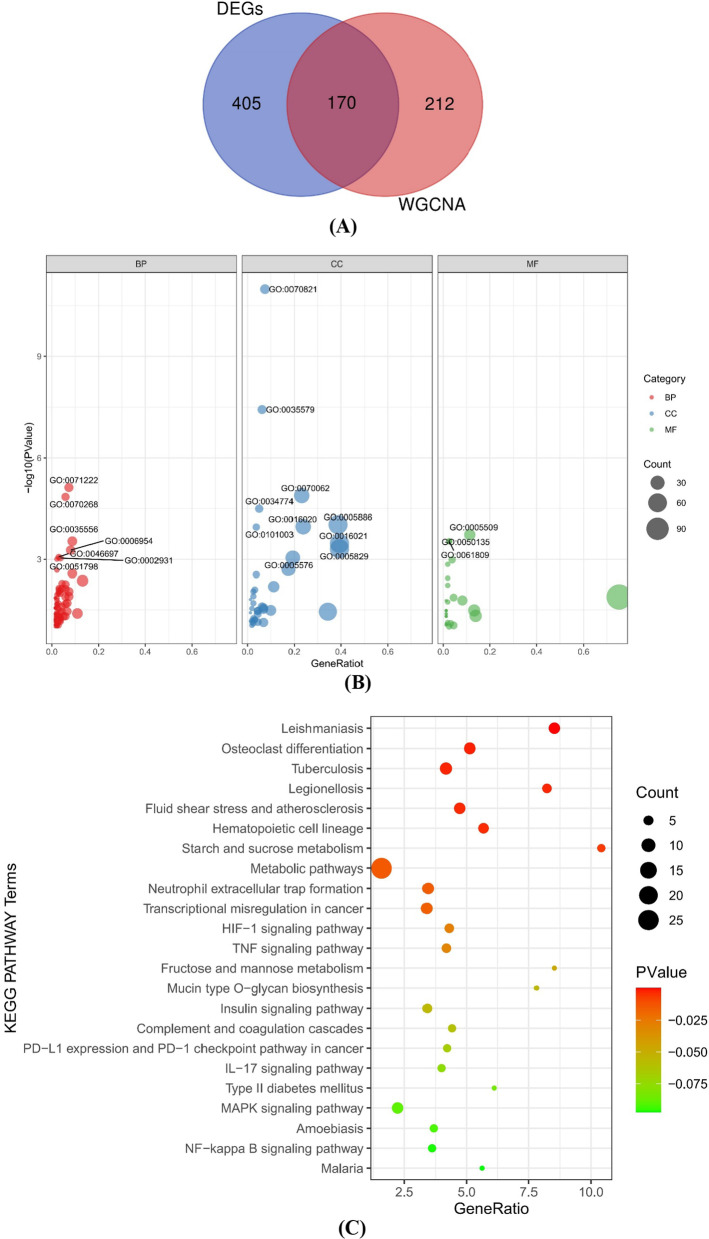
GO and KEGG analysis of 170 sepsis-related genes. **A** Venn diagram showing 170 sepsis-related genes obtained by DEGs and WGCNA. **B** All terms of GO categories of biological process (red), cellular component (blue) and molecular function (green). **C** KEGG pathway analyses of 170 genes

We identified 170 sepsis-related genes (Additional file 1: Table S1) based on the sepsis-related module and DEGs (Fig. 4A). GO terms for 170 genes fall into three categories: biological processes (BP), cellular components (CC), and molecular functions (MF) (Fig. 4B). The results of GO analysis were mainly associated with inflammation, cornification, and granule membrane, such as cellular response to lipopolysaccharide,, tertiary granule membrane, extracellular space and NAD + nucleotidase, cyclic ADP-ribose generating (Additional file 2 Table S2). In addition, we carried out a KEGG pathway enrichment analysis on 170 genes (Fig. 4C). The results of the enrichment analysis consistent with FDR < 0.05 was leishmaniasis. Detailed results of the KEGG analysis are shown in Additional file 2: Table S2. These results were positive for the present study and contribute to further research (Additional files 3, 4).

### Identification and analysis of hub genes in sepsis by the PPI network

**Fig. 5:**
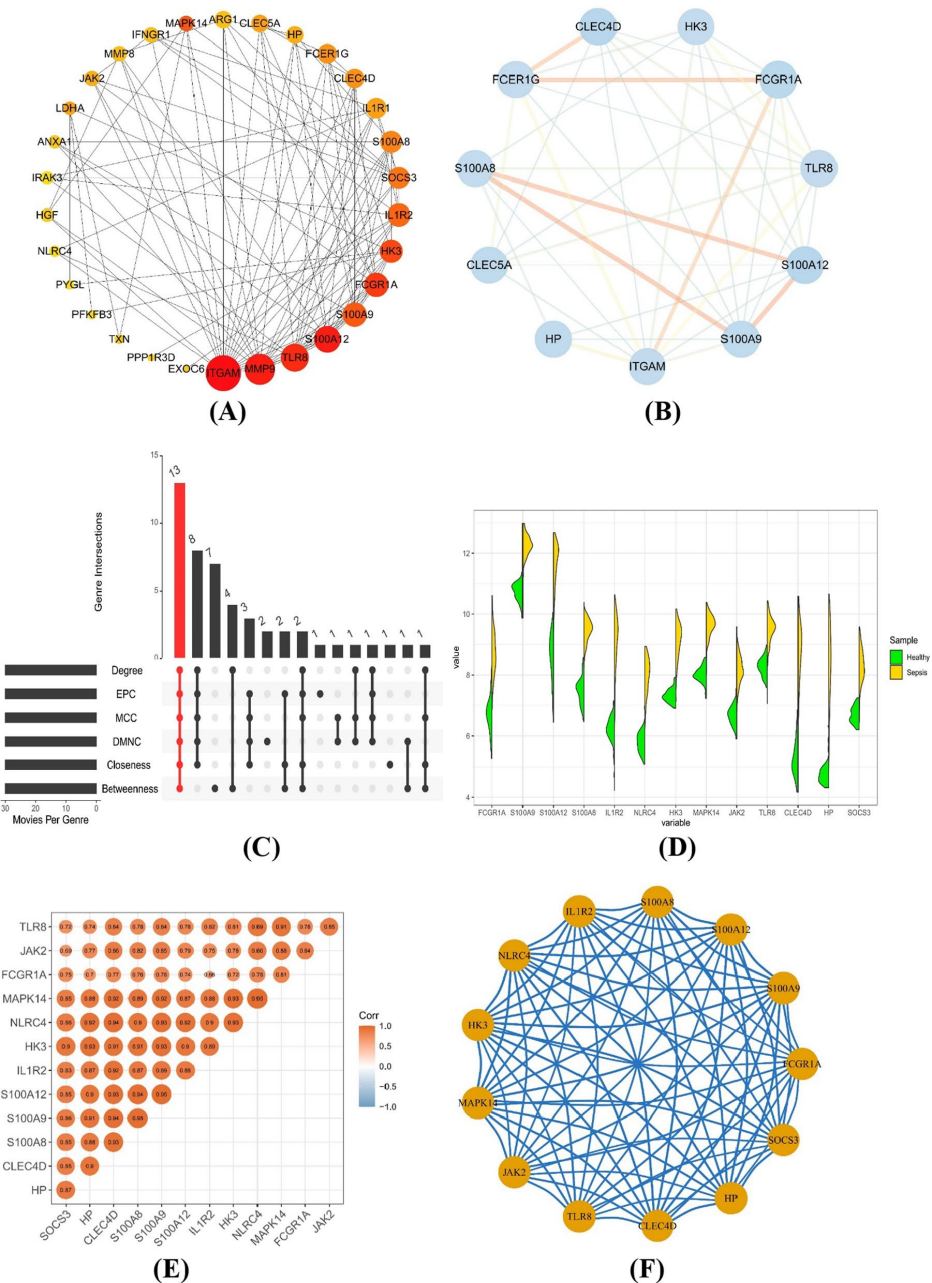
Identification and analysis of hub genes by the PPI network. **A** The top 30 genes of the degree algorithm of the PPI network. **B** The most insignificant module of the PPI network. **C** The hub genes were identified by six algorithms (Degree, EPC, MCC, DMNC, Closeness, and Betweenness). **D** Violin plot of hub gene expression. **E** Correlation heatmap of hub genes. **F** Correlation network map of hub genes

We acquired the PPI network with an interaction score of 0.400 based on the STRING database, including 163 nodes and 226 edges (Additional files 5, 6, 7, 8, 9, 10). We applied six algorithms (Degree, EPC, MCC, DMNC, Closeness, Betweenness) to mine 13 hub genes from the PPI network (Additional file 3: Table S3), which was the intersection of the top 30 genes of each algorithm (Fig. 5C). Figure 5A displays the network map of the top 30 genes of the degree algorithm. According to the MCODE plugin, the most insignificant module of the PPI network is shown in Fig. 5B. The expression analysis revealed that all 13 genes were expressed at higher levels in sepsis samples than in healthy samples (Fig. 5D). We calculated the correlations among hub genes, and the results demonstrated that they all had significant positive correlations (Fig. 5E). Meanwhile, the correlation network diagram also proved the tightness of the contact among them (Fig. 5F).

### Enrichment analysis of hub genes

**Fig. 6:**
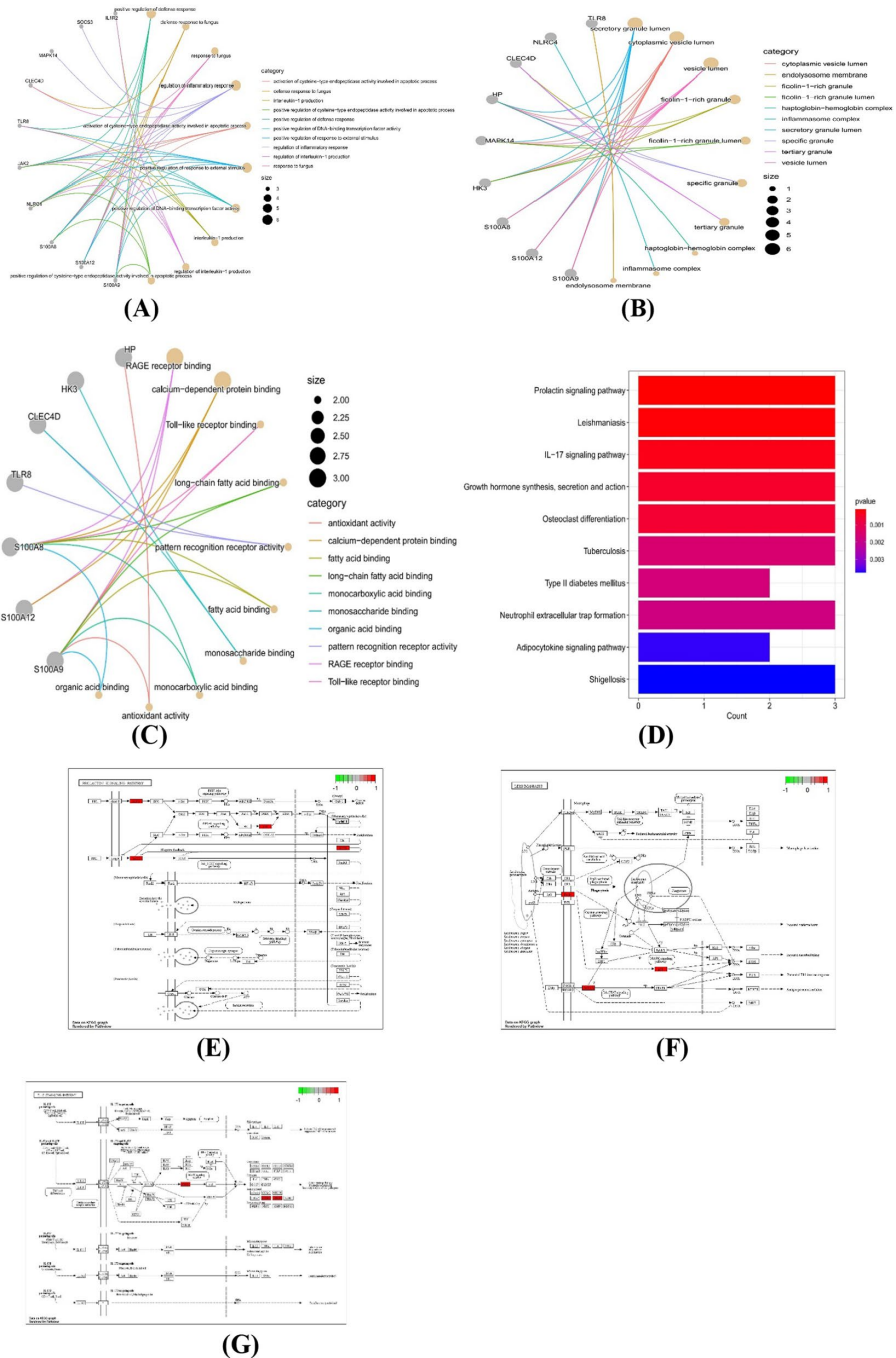
GO and KEGG analysis of hub genes. **A–C** GO enrichment analysis of hub genes (**A**: BP, **B**: CC, **C**: MF). The size of the node respondents for the number of gene counts. (**D**) KEGG enrichment analysis of hub genes; the colour of the bar represents the P value. (**E**–**G**) Prolactin signalling pathway, leishmaniasis, and IL-17 signalling pathway. Red indicates high expression in the pathway, and green indicates low expression in the pathway

To further investigate the connection of sepsis development with hub genes, we performed GO and KEGG enrichment analyses. GO enrichment analysis indicated that hub genes were focused on defence response, inflammation regulation and multiple receptor activation (Fig. 6A–C). According to KEGG analysis, the hub genes were involved in various signalling pathways, including the prolactin signalling pathway, leishmaniasis, the IL-17 signalling pathway, growth hormone synthesis, secretion and action (Fig. 6D–G) [12–14]. Detailed results of the enrichment analysis are shown in Additional file 2: Table S2. These results confirm the high association between hub genes and sepsis, as well as the apparent variation of hub genes in various immune and inflammatory conditions.

### Correlation of sepsis and pyroptosis based on subtype clustering

**Fig. 7:**
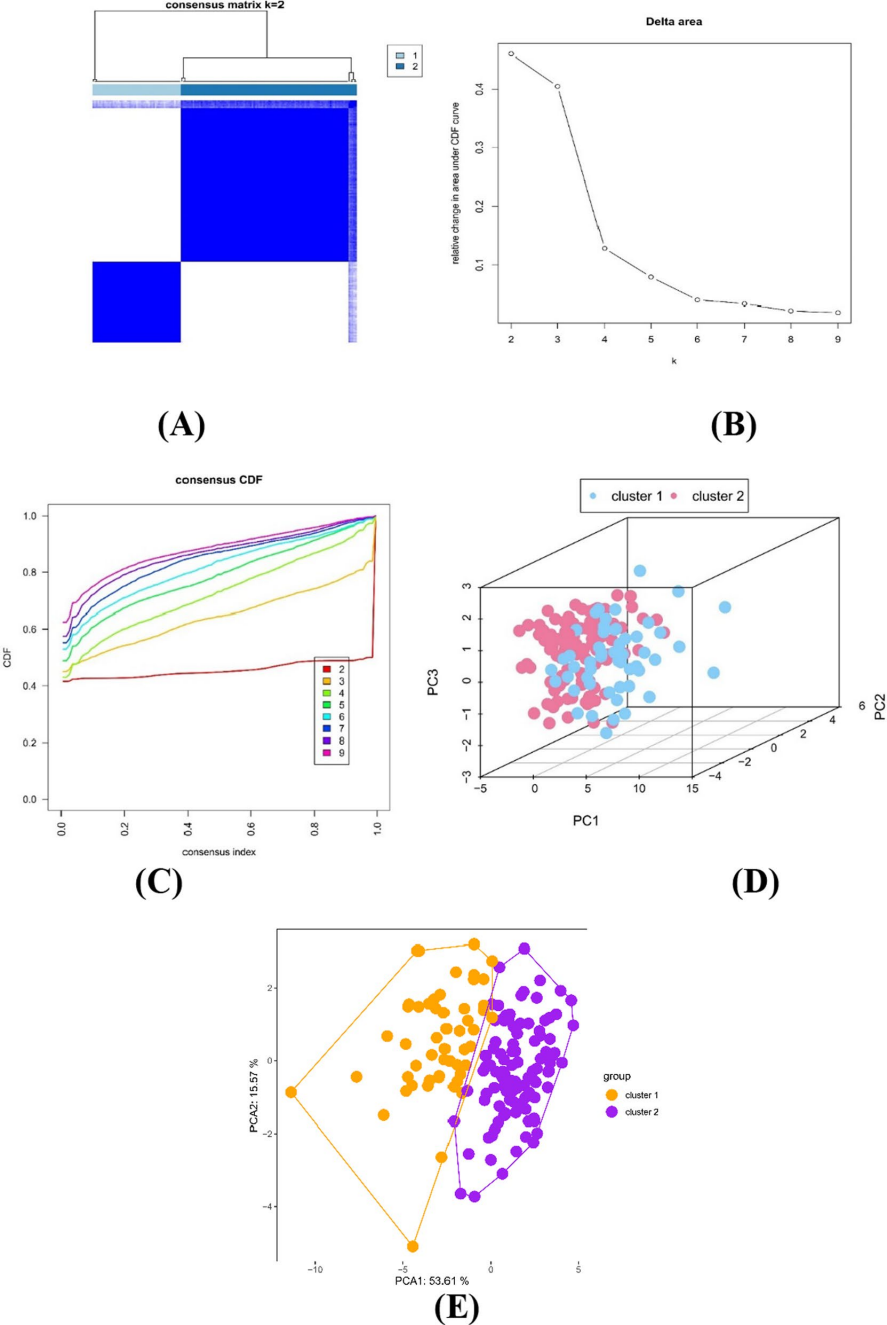
Identification of consensus clusters by pyroptosis-related genes. **A** When k = 2, there is a correlation between groups. **B** Relative change in the area under the cumulative distribution function (CDF) curve for k values from 2 to 9. **C** Consensus clustering CDF when the k value ranges from 2 to 9. **D** PCA of pyroptosis-related genes in the sepsis samples (Cluster 1 is marked in blue, and Cluster 2 is marked in red). **E** PCA of pyroptosis-related genes in the sepsis samples (Cluster 1 is marked in orange, and Cluster 2 is marked in purple)

Based on 33 PRGs provided by prior reviews, subtype analysis of sepsis was performed. According to Fig. 7B and 7C, k = 2 or k = 3 values would be acceptable; however, after dividing the samples into 3 groups, some data could not be well clustered; therefore, we decided to separate our data into 2 groups. The data could be well clustered when k = 2 (k: clustering variable) based on Figs. 7B and 7C. The matrix shown in Fig. 7A represents the consensus for k = 2 and indicates a well-defined two-block structure. As shown Fig. 7D and E, 33 PRGs could distinguish Cluster 1 from Cluster 2 from two different perspective, and we concluded that grouping by pyroptosis-related genes of sepsis expression was appropriate (k = 2). Thus, a possible correlation between pyroptosis-related genes and sepsis may also be demonstrated.

### Analysis and screening PRGs

**Fig. 8:**
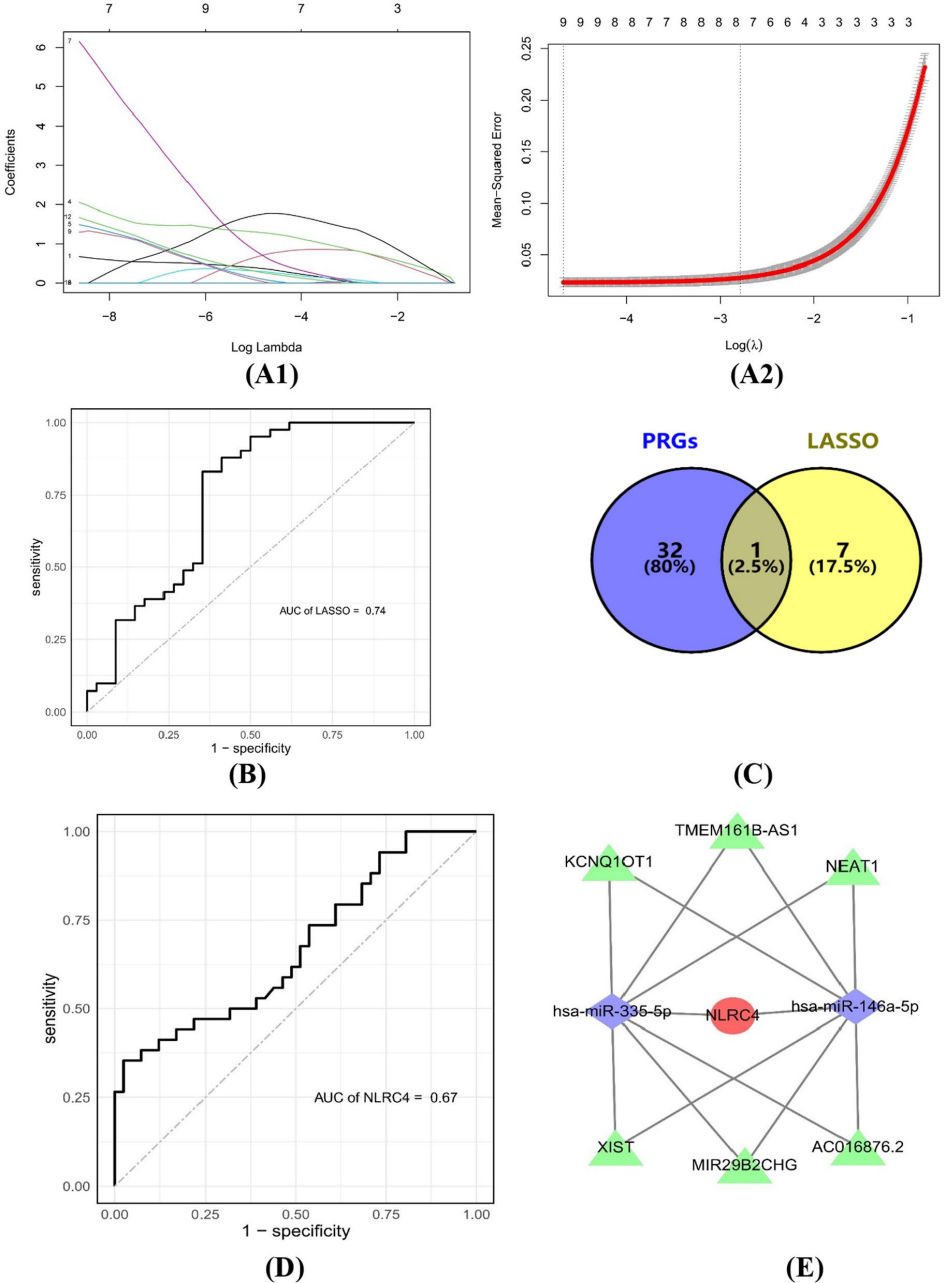
Analysis and screening of PRGs associated with sepsis. **A** Eight sepsis-related key genes obtained using the LASSO algorithm. **B** Application of an external dataset to validate the predictive model. **C** The PRG mostly associated with sepsis was identified by a predictive model, the GeneCards database and prior reviews. **D** Applying an external dataset to validate the PRG mostly associated with sepsis. **E** Construction of the ceRNA network around the PRG mostly associated with sepsis

We yielded 8 key genes by applying validated machine learning algorithms (LASSO) from 13 hub genes (Fig. 8A1, A2). We used GSE32707, as an external dataset, to evaluate the efficiency of the supervised machine learning algorithms using ROC curves (Fig. 8B). The AUC value of LASSO was 0.74, and we considered it the optimal sepsis prediction model. According to the 33 PRGs provided by prior reviews, only NLRC4 was associated with pyroptosis in the 8 key genes related to sepsis (Fig. 8C). Finally, ROC curves were plotted based on the external validation dataset (GSE32707) to verify the potential value of NLRC4 as an early diagnostic marker or therapeutic target for sepsis patients. The AUC value of NLRC4 was 0.67, which was greater than or equal to 0.65, and it was identified as a sepsis-related key gene (Fig. 8D). To explore the upstream targets of PRGs associated with sepsis, we used the NetworkAnalyst online tool to predict the miRNAs of NLRC4. The StarBase database (https://starbase.sysu.edu.cn/) was employed to predict lncRNAs based on hsa-miR-335-5p and hsa-miR-146a-5p, as well as to construct a ceRNA network with 1 mRNA (NLRC4), 2 miRNAs (hsa-miR-335-5p, hsa-miR-146a-5p) and 6 lncRNAs (MIR29B2CHG, TMEM161B-AS1, KCNQ1OT1, NEAT1, AC016876.2, XIST) (Fig. 8E).

## Discussion

Sepsis is currently one of the major global health burdens and the leading cause of death for patients in intensive care units (ICUs) [15]. Therefore, there is an urgent need to find a therapeutic target that can be used as an early diagnostic or effective treatment target to improve diagnostic efficiency and patient prognosis and quality of life. In this study, we first screened 170 genes associated with sepsis by WGCNA and differential expression analysis, which resulted in the identification of 13 genes closely connected to sepsis. The results of functional enrichment analysis suggested that these genes were mainly involved in the regulation of the inflammatory response and the positive regulation of bacterial and fungal defence responses, all of which indicated an association with the pathogenesis and course of sepsis. Therefore, these findings could provide a strong theoretical basis for further related studies of the 13 genes and enhance the validity of the results.

Pyroptosis is a mode of programmed cell death that is distinguished from apoptosis and could be involved in the innate immune response of the body, activation of immune cell phagocytosis and clearance of pathogens [16, 17]. During sepsis pathogenesis, an inappropriate or excessive inflammatory response of the body may cause secondary infection or even organ failure [18]. Correspondingly, excessive pyroptosis could also lead to an uncontrollable inflammatory response, resulting in a poor prognosis [19, 20]. With the improvement of scientific research, a growing number of studies have attempted to elucidate the mutual relationship existing for the pathogenesis of sepsis and pyroptosis. It has been shown that caspase-1 activated by LPS can act on the pannexin-1 and P2X7 signalling pathways to induce scorch production and severe inflammatory responses, and this could be a potential target for the treatment of gram-negative bacterial sepsis [21]. Additional studies have demonstrated that downregulated miR-21 could suppress cystein-1 activation and GSDMD cleavage, acting through protein A20 to regulate the nuclear factor kappa B (NF-kB) pathway, thus serving as an essential positive regulator of pyroptosis and septic shock [22, 23]. Therefore, further exploration of the role played by pyroptosis in the pathogenesis of sepsis may provide novel potential therapeutic targets for sepsis. Machine learning, a well-established technology in the biomedical field, plays an irreplaceable role in improving the efficiency of clinical diagnosis and providing the best treatment options. We applied machine learning algorithms combined with relevant reviews and databases to screen for PRGs associated with sepsis and ultimately identified NLRC4 as a potentially effective therapeutic target for sepsis.

The NOD-like receptor (NLR) family, CARD domain-containing protein 4 (NLRC4), was initially described as a pro-apoptotic protein and demonstrated to detect cytosolic flagellin [24–26]. NLRC4, a pivotal component of the inflammasome, is involved in endogenous danger signalling responses to multiple microbial spines and macrophage scorching [27]. Recruitment of the NLRC4 inflammasome may have a substantial effect on gram-negative bacterial infections, especially those associated with Salmonella typhimurium [28]. It has been reported that overexpression of NLRC4 increases macrophage inflammasome activity, leading to infantile small bowel colitis syndrome and recurrent macrophage activation syndrome [29, 30]. In addition, another study found that decreased NLRC4 reduced the inflammatory response; during gram-positive pneumonia, NLRC4 knockdown mice exhibited reduced inflammation and controlled bacteria more effectively than wild-type infected mice [30, 31]. Pyroptosis is a proinflammatory form of regulated cell death dependent on caspase-1 activation [5]. When in recognition of danger or pathogen-associated molecular patterns, the inflammasome initiation sensor (NLRC4) activates caspase-1, which is considered the typical inflammatory vesicle activation mode [32]. In this study, NLRC4 was highly expressed in all patients with sepsis; therefore, it is reasonable to believe that NLRC4 may cause pyroptosis by activating caspase-1 and promoting the inflammatory response, which consequently leads to the development of sepsis. As a result, NLRC4 may be considered a potential therapeutic target of sepsis for further research. At present, some lncRNAs have been demonstrated to act as important regulators in the pathogenesis of sepsis [33]. For example, it was reported that there were significant differences in the expression of lncRNA ENST00000504301.1 and ENST00000452391.1 between sepsis survivors and nonsurvivors [34, 35]. To further explore the impact of a pyroptosis gene (NLRC4) on sepsis at a deeper level, we predicted the upstream targeting factor miRNAs and lncRNAs and constructed a ceRNA network of 6 lncRNAs (MIR29B2CHG, TMEM161B-AS1, KCNQ1OT1, NEAT1, AC016876.2, XIST) and 2 miRNAs (hsa-miR-335-5p, hsa-miR-146a-5p) around NLRC4.

Meanwhile, there are limitations to this study. The prediction results of lncRNAs and miRNAs are in a wide range and require more experimental data and literature to corroborate. Additionally, the PRGs identified in this study that have the potential to be therapeutic targets for sepsis require further literature support and basic experimental validation.

## Conclusions

In this study, we identified NLRC4 as a PRG associated with sepsis based on machine learning and constructed a ceRNA network of lncRNAs and miRNAs around NLRC4, which may serve as early molecular biomarkers for therapeutic targets of sepsis. In the future, these molecular markers deserve further study in follow-up and require additional datasets and further experimental validation at the cellular or specimen level.

## Supplementary information

**Additional file 1. Table S1:**The details of the DEGs, WGCNA, and intersection of them.

**Additional file 2**. **Table S2**: The detailed results of the GO and KEGG.

**Additional file 3. Table S3**: The genes of six algorithms (Degree, EPC, MCC, DMNC, Closeness, Betweenness).

**Additional file 4. Table S4**: The details of PRGs.

**Additional file 5: Figure S1**: The PPI network.

**Additional file 6: Figure S2**: The subnetwork of PPI (Betweenness_top30).

**Additional file 7: Figure S3**Figure S3: The subnetwork of PPI (Closeness_top30).

**Additional file 8: Figure S4**: The subnetwork of PPI (DMNC_top30).

**Additional file 9: Figure S5**: The subnetwork of PPI (EPC_top30).

**Additional file 10: Figure S6**: The subnetwork of PPI (MCC_top30).

## Data Availability

Publicly available datasets were analysed in this study. The datasets (GSE134347 and GSE32707) analysed during the current study are available in the Gene Expression Omnibus (GEO) (https://www.ncbi.nlm.nih.gov/geo/) and GeneCards databases (https://www.genecards.org/).

## Abbreviations

WGCNA: Weighted gene coexpression network analysis
PPI: Protein‒protein interaction
PRGs: Pyroptosis-related genes
ROC: Receiver operating characteristic
lncRNAs: Long noncoding RNAs
mRNAs: Messenger RNAs
GEO: Gene Expression Omnibus
DEGs: Differentially expressed genes
TOM: Topological overlap matrix
GO: Gene ontology
KEGG: Kyoto Encyclopedia of Genes and Genomes
DAVID: Database for annotation, visualization, and integrated discovery
STRING: Database for annotation, visualization, and integrated discovery
CDF: Cumulative distribution function
PCA: Principal component analysis
LASSO: Least absolute shrinkage and selection operator
MS: Modular significance
GS: Gene significance
BP: Biological processes
MF: Molecular functions
CC: Cellular components
ICUs: Intensive care units
NF-kB: Nuclear factor kappa B
CNS: Central nervous system
NLR: NOD-like receptor
NLRC4: NOD-like receptor (NLR) family, CARD domain-containing protein 4

## References

[1] Dellinger RP, Levy MM, Rhodes A, Annane D, Gerlach H, Opal SM (2013). Surviving sepsis campaign: international guidelines for management of severe sepsis and septic shock, 2012. Intensive Care Med.

[2] Rhodes A, Evans LE, Alhazzani W, Levy MM, Antonelli M, Ferrer R (2017). Surviving sepsis campaign: international guidelines for management of sepsis and septic shock: 2016. Crit Care Med.

[3] Seymour CW, Gesten F, Prescott HC, Friedrich ME, Iwashyna TJ, Phillips GS (2017). Time to treatment and mortality during mandated emergency care for sepsis. N Engl J Med.

[4] Bertheloot D, Latz E, Franklin BS (2021). Necroptosis, pyroptosis and apoptosis: an intricate game of cell death. Cell Mol Immunol.

[5] Vande Walle L, Lamkanfi M (2016). Pyroptosis. Curr Biol.

[6] Aglietti RA, Dueber EC (2017). Recent insights into the molecular mechanisms underlying pyroptosis and gasdermin family functions. Trends Immunol.

[7] Khorkova O, Hsiao J, Wahlestedt C (2015). Basic biology and therapeutic implications of lncRNA. Adv Drug Deliv Rev.

[8] Zhang T-N, Li D, Xia J, Wu Q-J, Wen R, Yang N (2017). Non-coding RNA: a potential biomarker and therapeutic target for sepsis. Oncotarget.

[9] Safran M, Rosen N, Twik M, BarShir R, Stein TI, Dahary D, Abugessaisa I, Kasukawa T (2021). The GeneCards Suite. Practical guide to life science databases.

[10] Langfelder P, Horvath S (2008). WGCNA: an R package for weighted correlation network analysis. BMC Bioinform.

[11] Huang DW, Sherman BT, Lempicki RA (2009). Systematic and integrative analysis of large gene lists using DAVID bioinformatics resources. Nat Protoc.

[12] Kanehisa M, Goto S (2000). KEGG: kyoto encyclopedia of genes and genomes. Nucleic Acids Res.

[13] Kanehisa M (2019). Toward understanding the origin and evolution of cellular organisms. Protein Sci.

[14] Kanehisa M, Furumichi M, Sato Y, Kawashima M, Ishiguro-Watanabe M (2022). KEGG for taxonomy-based analysis of pathways and genomes. Nucleic Acids Res.

[15] Keeley A, Hine P, Nsutebu E (2017). The recognition and management of sepsis and septic shock: a guide for non-intensivists. Postgrad Med J.

[16] Miao EA, Leaf IA, Treuting PM, Mao DP, Dors M, Sarkar A (2010). Caspase-1-induced pyroptosis is an innate immune effector mechanism against intracellular bacteria. Nat Immunol.

[17] Aachoui Y, Leaf IA, Hagar JA, Fontana MF, Campos CG, Zak DE (2013). Caspase-11 protects against bacteria that escape the vacuole. Science.

[18] Kaukonen K-M, Bailey M, Pilcher D, Cooper DJ, Bellomo R (2015). Systemic inflammatory response syndrome criteria in defining severe sepsis. N Engl J Med.

[19] Pu Q, Gan C, Li R, Li Y, Tan S, Li X (2017). Atg7 deficiency intensifies inflammasome activation and pyroptosis in sepsis. J Immunol.

[20] Pfalzgraff A, Heinbockel L, Su Q, Brandenburg K, Weindl G (2017). Synthetic anti-endotoxin peptides inhibit cytoplasmic LPS-mediated responses. Biochem Pharmacol.

[21] Yang D, He Y, Muñoz-Planillo R, Liu Q, Núñez G (2015). Caspase-11 requires the pannexin-1 channel and the purinergic P2X7 pore to mediate pyroptosis and endotoxic shock. Immunity.

[22] Xue Z, Xi Q, Liu H, Guo X, Zhang J, Zhang Z (2019). miR-21 promotes NLRP3 inflammasome activation to mediate pyroptosis and endotoxic shock. Cell Death Dis.

[23] Zheng X, Chen W, Gong F, Chen Y, Chen E (2021). The role and mechanism of pyroptosis and potential therapeutic targets in sepsis: a review. Front Immunol.

[24] Geddes BJ, Wang L, Huang WJ, Lavellee M, Manji GA, Brown M (2001). Human CARD12 is a novel CED4/Apaf-1 family member that induces apoptosis. Biochem Biophys Res Commun.

[25] Franchi L, Amer A, Body-Malapel M, Kanneganti T-D, Ozören N, Jagirdar R (2006). Cytosolic flagellin requires Ipaf for activation of caspase-1 and interleukin 1beta in salmonella-infected macrophages. Nat Immunol.

[26] Miao EA, Alpuche-Aranda CM, Dors M, Clark AE, Bader MW, Miller SI (2006). Cytoplasmic flagellin activates caspase-1 and secretion of interleukin 1beta via Ipaf. Nat Immunol.

[27] Kofoed EM, Vance RE (2012). NAIPs: building an innate immune barrier against bacterial pathogens. NAIPs function as sensors that initiate innate immunity by detection of bacterial proteins in the host cell cytosol. BioEssays.

[28] Sundaram B, Kanneganti T-D (2021). Advances in understanding activation and function of the NLRC4 inflammasome. Int J Mol Sci.

[29] Canna SW, de Jesus AA, Gouni S, Brooks SR, Marrero B, Liu Y (2014). An activating NLRC4 inflammasome mutation causes autoinflammation with recurrent macrophage activation syndrome. Nat Genet.

[30] Romberg N, Al Moussawi K, Nelson-Williams C, Stiegler AL, Loring E, Choi M (2014). Mutation of NLRC4 causes a syndrome of enterocolitis and autoinflammation. Nat Genet.

[31] Paudel S, Ghimire L, Jin L, Baral P, Cai S, Jeyaseelan S (2019). NLRC4 suppresses IL-17A-mediated neutrophil-dependent host defense through upregulation of IL-18 and induction of necroptosis during Gram-positive pneumonia. Mucosal Immunol.

[32] Man SM, Karki R, Kanneganti T-D (2017). Molecular mechanisms and functions of pyroptosis, inflammatory caspases and inflammasomes in infectious diseases. Immunol Rev.

[33] Fang Y, Hu J, Wang Z, Zong H, Zhang L, Zhang R (2018). LncRNA H19 functions as an Aquaporin 1 competitive endogenous RNA to regulate microRNA-874 expression in LPS sepsis. Biomed Pharmacother.

[34] Dai Y, Liang Z, Li Y, Li C, Chen L (2017). Circulating long noncoding RNAs as potential biomarkers of sepsis: a preliminary study. Genet Test Mol Biomarkers.

[35] Li Y, Li Y, Bai Z, Pan J, Wang J, Fang F (2017). Identification of potential transcriptomic markers in developing pediatric sepsis: a weighted gene co-expression network analysis and a case-control validation study. J Transl Med.

