# Supplementary figures and images for: Revealing novel pyroptosis-related therapeutic targets for sepsis based on machine learning
Source: BMC Med Genomics. 2023 Feb 10;16:23. doi: 10.1186/s12920-023-01453-7 (PMC9912626; doi:10.1186/s12920-023-01453-7)

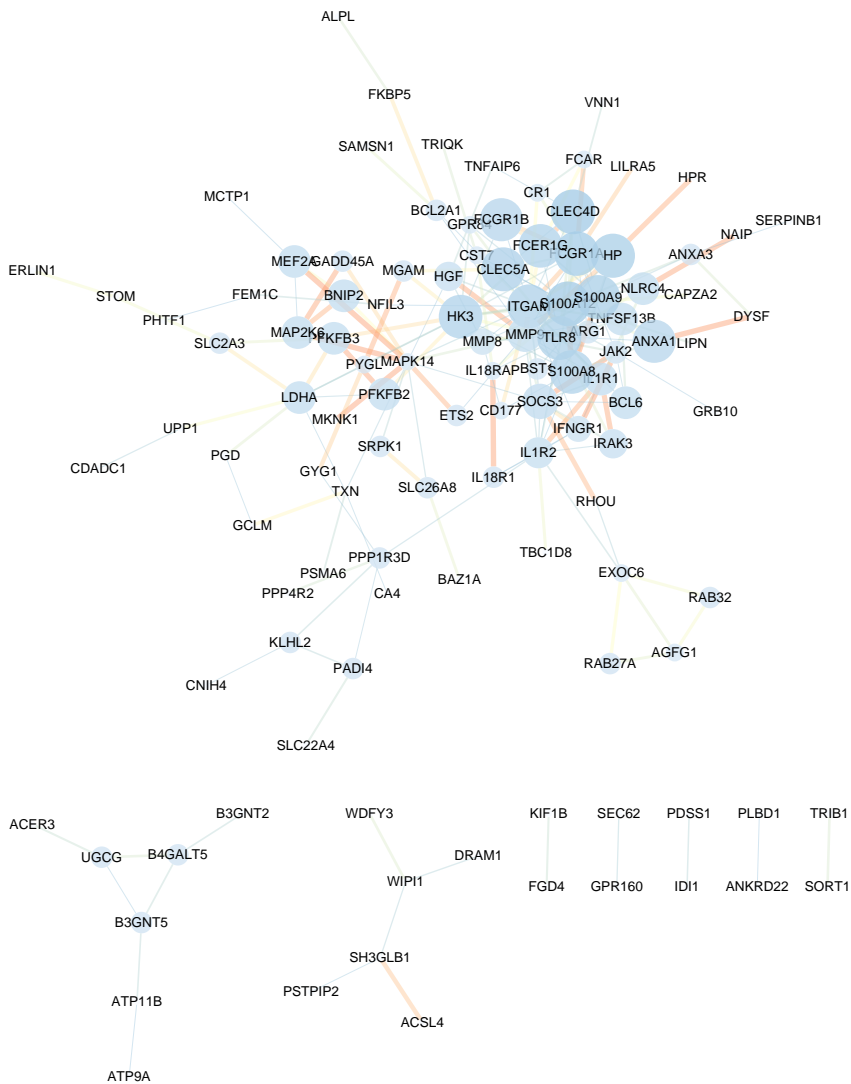

Supplement: Supplementary file 5 — Additional file 5: Figure S1: The PPI network. [file 12920_2023_1453_MOESM5_ESM.pdf]

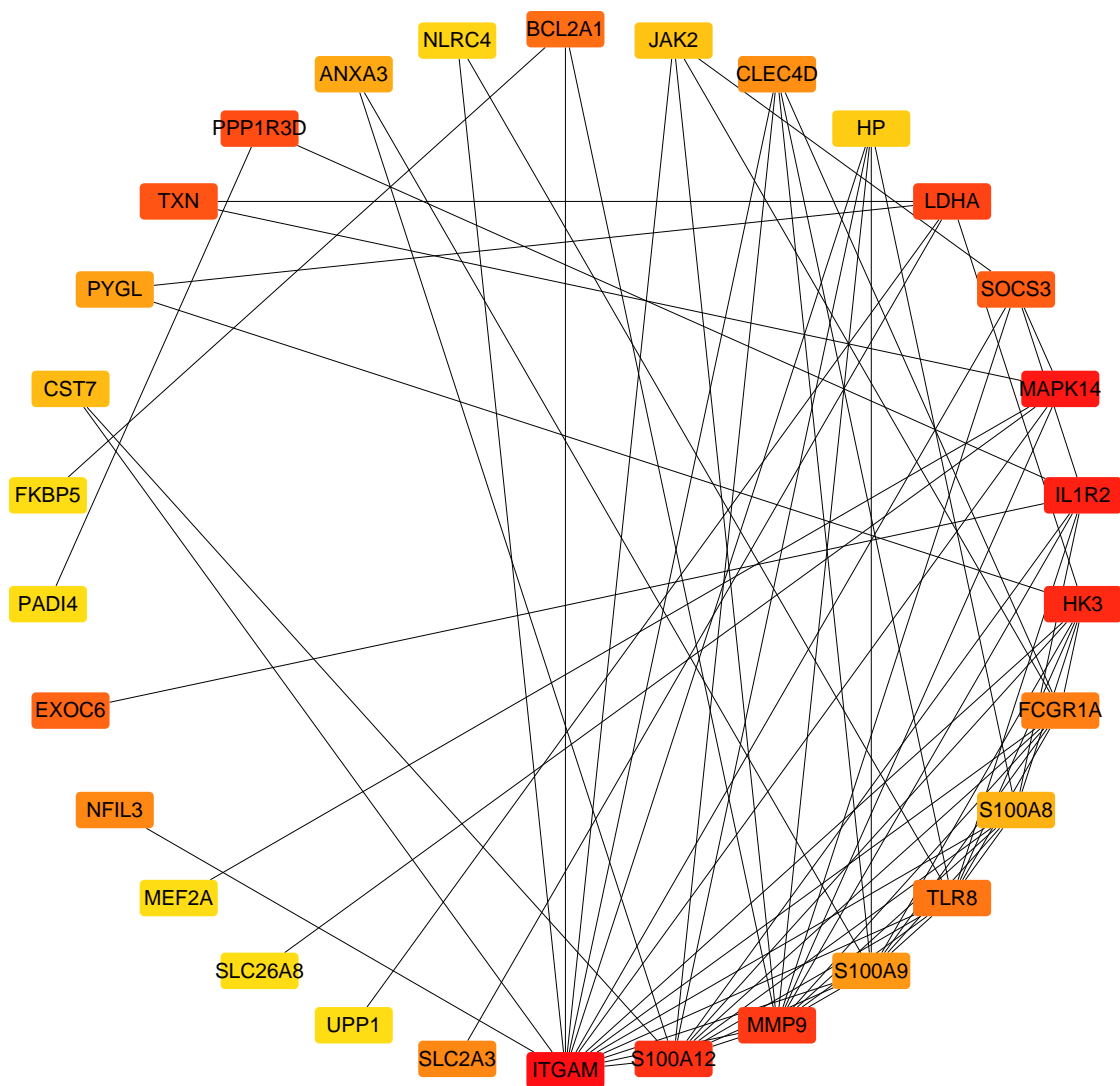

Supplement: Supplementary file 6 — Additional file 6: Figure S2: The subnetwork of PPI (Betweenness_top30). [file 12920_2023_1453_MOESM6_ESM.pdf]

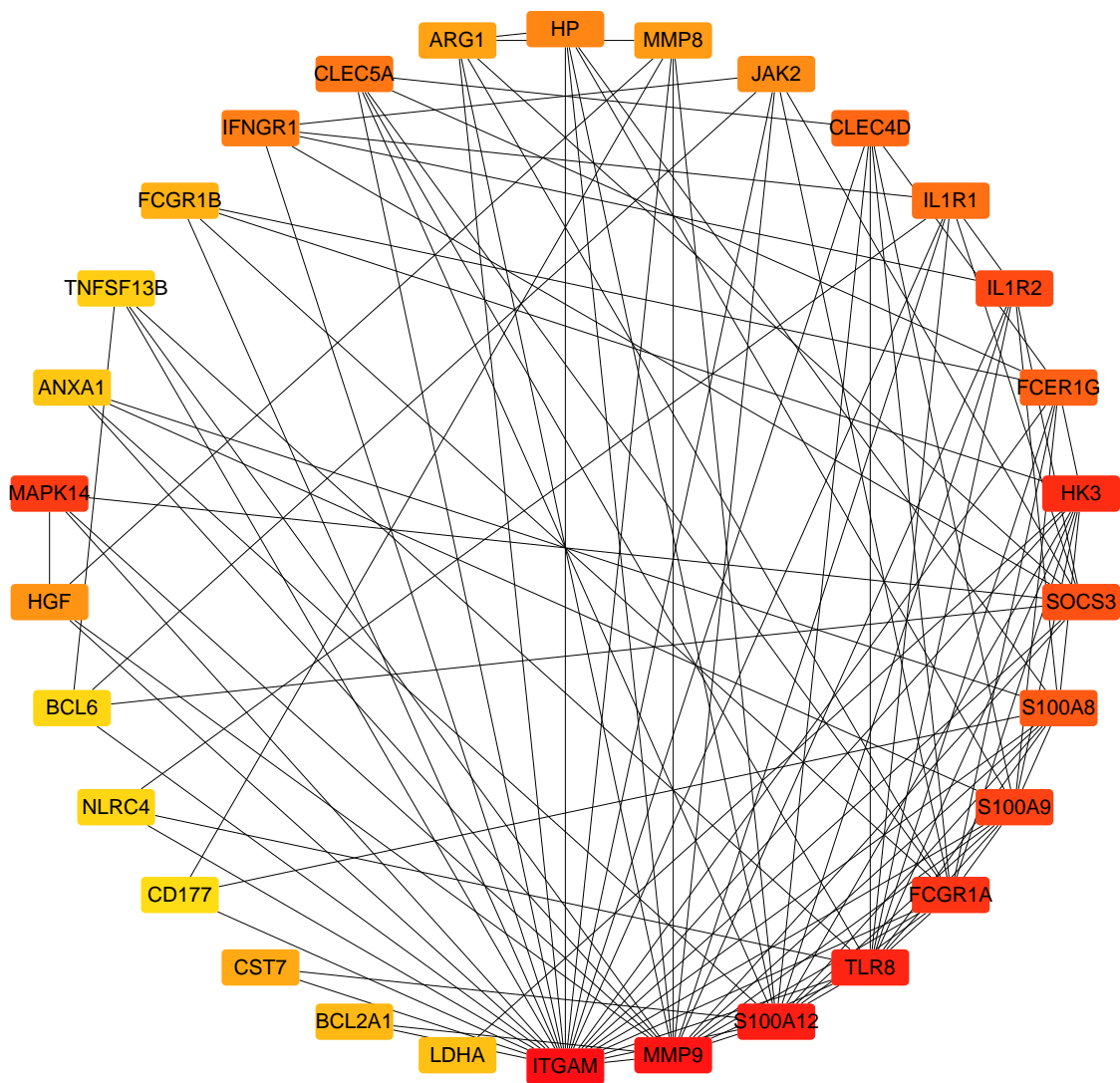

Supplement: Supplementary file 7 — Additional file 7: Figure S3Figure S3: The subnetwork of PPI (Closeness_top30). [file 12920_2023_1453_MOESM7_ESM.pdf]

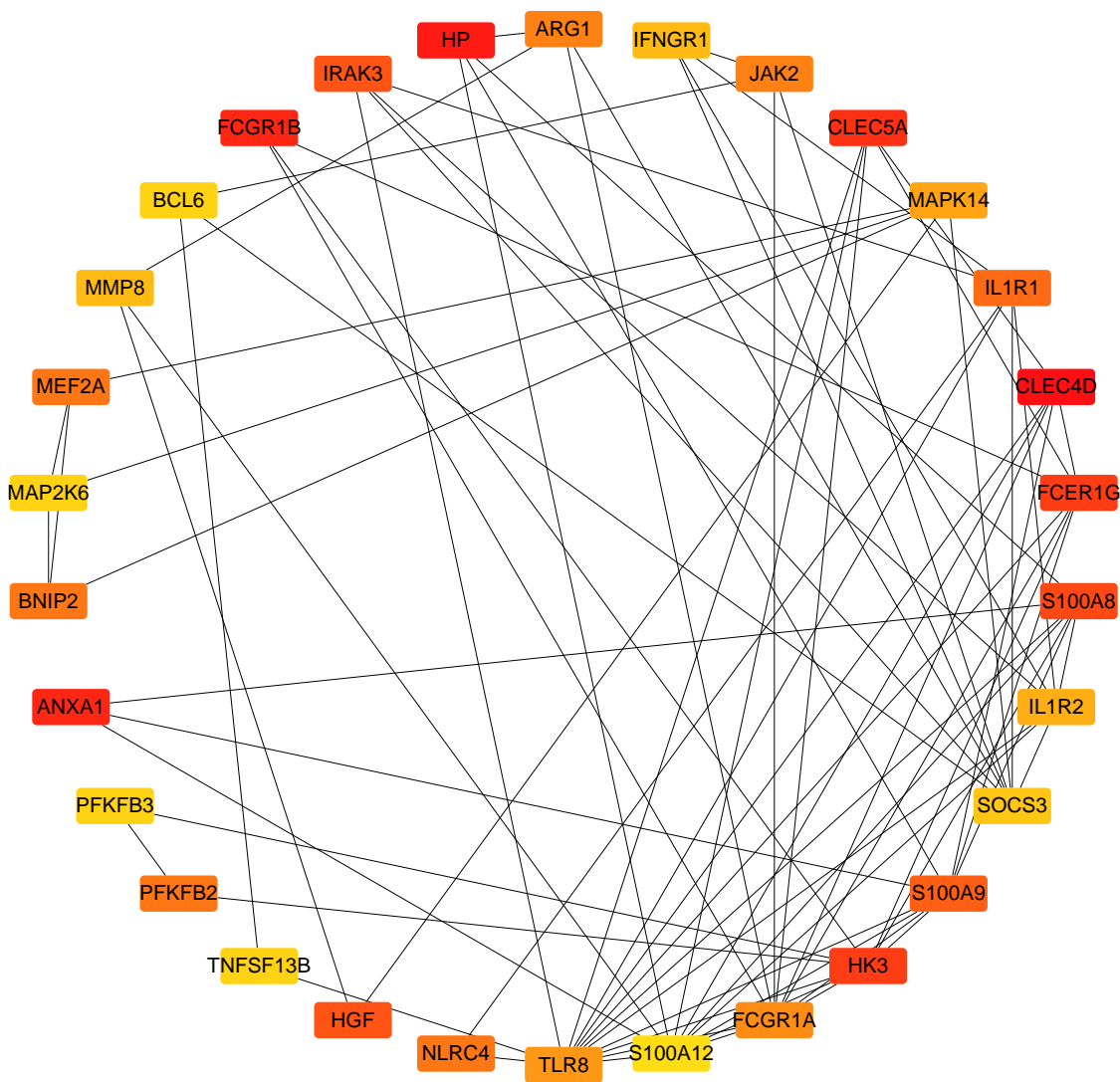

Supplement: Supplementary file 8 — Additional file 8: Figure S4: The subnetwork of PPI (DMNC_top30). [file 12920_2023_1453_MOESM8_ESM.pdf]

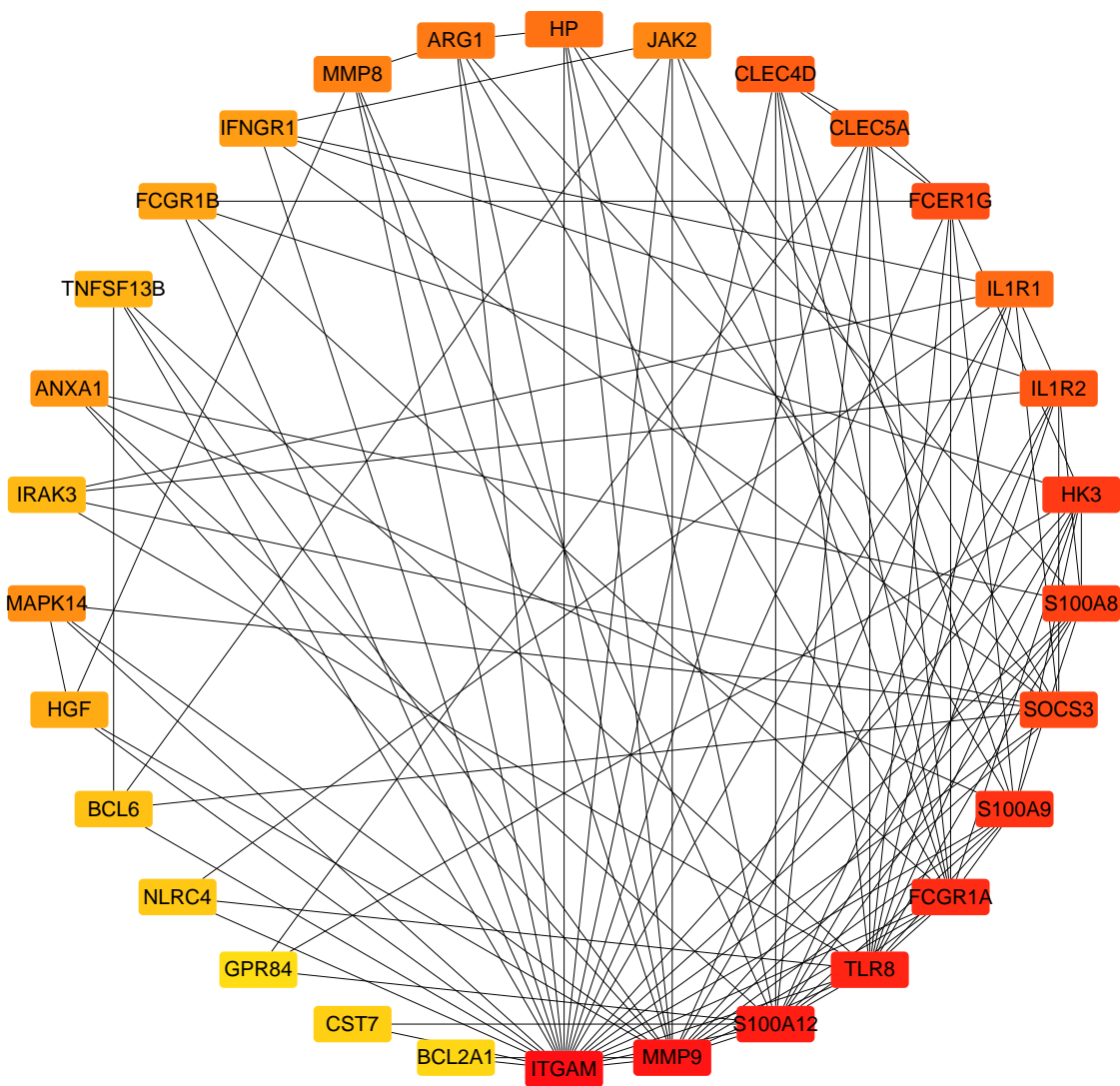

Supplement: Supplementary file 9 — Additional file 9: Figure S5: The subnetwork of PPI (EPC_top30). [file 12920_2023_1453_MOESM9_ESM.pdf]

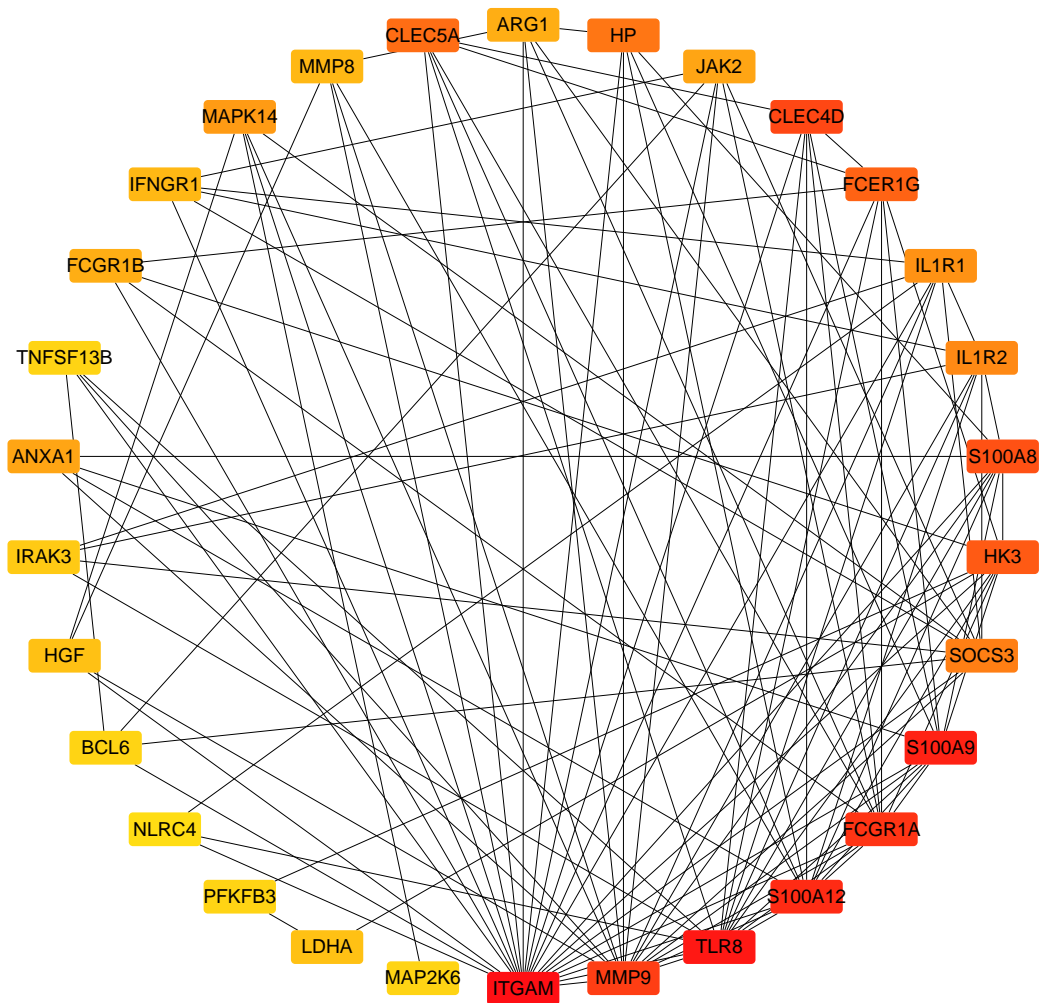

Supplement: Supplementary file 10 — Additional file 10: Figure S6: The subnetwork of PPI (MCC_top30). [file 12920_2023_1453_MOESM10_ESM.pdf]
